# Supplementary material for: Oxidative Stress Markers in Multiple Sclerosis
Source: Int J Mol Sci. 2024 Jun 7;25(12):6289. doi: 10.3390/ijms25126289 (PMC11203935; doi:10.3390/ijms25126289)
Supplement: Supplementary file 1 [file ijms-25-06289-s001.zip › ijms-2872596-Table captions.pdf]

International Journal of *Molecular Sciences*

MDPI

Table S1. Oxidative stress markers in brain/spinal cord, cerebrospinal fluid, blood cells, serum, plasma, urine, and saliva from multiple sclerosis patients and healthy controls. BACE1: β-site amyloid precursor protein-cleaving enzyme 1; CDMS: clinically defined MS; CIS: clinically isolated syndrome; CSF: cerebrospinal fluid, DNA: deoxyribonucleic acid; EDSS: expanded disability status scale; ER: endoplasmic reticulum; F: female; FRAP: ferric-reducing antioxidant power; GADPH: glyceraldehydes 3-phosphate-dehydrogenase; GPe: globus pallidus externus; GPi: globus pallidus internus; GPx: glutathione peroxidase; GSH: glutathione; GSSG: oxidized glutathione; GSSG-R: glutathione reductase; GST: glutathione transferase; HC: healthy control; HMA: human mercaptoalbumin; ^{1}HMRS: proton magnetic resonance spectroscopy; HNA: 4-hydroxy-2-nonenal; HNA1/HNA2: human non-mercaptoalbumin 1 and 2; HO-1: heme oxygenase 1; has: human serum albumin; Hsp: heat shock protein; HSP-32: heat shock protein 32; IL: interleukin; M: male; MDA: malonyl dialdehyde; MPO: myeloperoxidase; MRI: magnetic resonances imaging; MS: multiple sclerosis; MTCO-1: cytochrome c oxidase subunit 1; NADPH: nicotinamide-adenine dinucleotide phosphate; NAGM: non-affected gray matter; NAWM: non-affected white matter; NOX1: NADPH oxidase; NGAL: neutrophil gelatinase-associated lipocalin; NIND: non-inflammatory neurological diseases; NO: nitric oxide; NOX-1: NADPH oxidase; NPH: normal pressure hydrocephalus; NQO1: NAD(P)H quinine oxidoreductase 1; Nrf2: NF-E2-related factor 2; 8-OH-dG: 8-hydroxy-deoxyguanosine; OND: other neurological diseases; OSI: oxidative stress index; PAR: polyADP ribose; PARP-1: polyADP ribose polymerase 1; PON: paraoxonase; PPMS: primary progressive MS; PRDX: peroxiredoxin; PREP: prolyoligopeptidase; ROS: radical oxygen species; RRMS: relapsing-remitting MS; SOD: superoxide-dismutase; SPMS: secondary progressive MS; sTFR: soluble transferrin receptor; TAS: total antioxidant status (capacity); TBA: thiobarbituric acid; TBARS: thiobarbituric acid reactive substances; TF: transferrin; TOS: total oxidant status (capacity); TRAP: total-reducing antioxidant power; Trx: thioredoxin, TrxR: thioredoxin receptor.

Table S2. Association studies regarding genetic polymorphisms in genes involved in oxidative stress and the risk for multiple sclerosis. CNV: copy number variations; F: female; EDSS: expanded disability status scale; HC: healthy controls; HRR: haplotype relative risk; M: male; MS: multiple sclerosis; RPMS: relapsing-progressive MS; RRMS: relapsing-remitting MS; SNV: single-nucleotide variations; SPMS: secondary progressive MS; TDT: transmission disequilibrium test; VNTR: variable number of tandem repeats.
